# Genetic association of Toll-like receptor 4 gene and coronary artery disease in a Chinese Han population

**DOI:** 10.1186/s40064-016-3177-2

**Published:** 2016-09-13

**Authors:** Liting Zhou, Dongchun Zheng, Shuyue Wang, Jian Zhu, Yiyang Jia, Di Sun, Jin Xu, Qi Wang, Huaiji Chen, Feng Xu, Bo Li, Lin Ye

**Affiliations:** 1 Department of Occupational and Environmental Health, School of Public Health, Jilin University, 1163 Xin Min Street, Changchun, 130021 China; 2 Department of Emergency, China-Japan Union Hospital, Jilin University, Changchun, China; 3 Department of Epidemiology and Statistics, School of Public Health, Jilin University, 1163 Xin Min Street, Changchun, 130021 China

**Keywords:** Coronary artery disease, Gene polymorphism, Toll-like receptor 4

## Abstract

**Purpose:**

Toll-like receptor 4 (TLR4) is known to be involved in innate immunity and inflammatory responses that play important roles in the pathogenesis of coronary artery disease (CAD). But the relationship between *TLR4* gene and CAD has yet to be investigated. The present study aimed to evaluate the association of *TLR4* gene polymorphisms with CAD susceptibility in a Chinese Han population.

**Methods:**

A total of 1094 subjects (577 unrelated patients with CAD and 517 controls) were recruited between 2008 and 2012. Three tag SNPs (rs1927907, rs1927911 and rs11536889) present in the *TLR4* gene were genotyped using Sequenom Mass-ARRAY system.

**Results:**

The genotypic distributions of the three SNPs were not deviate from Hardy–Weinberg equilibrium. There was no significant difference in distributions of allelic frequencies of each SNPs between healthy controls and CAD patients (*P* > 0.05). Genotype frequencies of *TLR4* gene did not show any statistically significant difference between the two groups in co-dominant, dominant or recessive genetic models (*P* > 0.05). The frequency of haplotypes in the case group was similar to that in the control group (*P* > 0.05).

**Conclusion:**

*TLR4* gene do not relate to genetic susceptibility of CAD in the Chinese Han population.

## Background

Coronary artery disease (CAD) is the leading cause of morbidity and mortality worldwide (Roger et al. 2012). As a complex disease, the main pathological features of CAD are atherosclerotic changes, which include vascular endothelial damage, adhesion and migration of monocytes, lipid accumulation in macrophages, and the formation of foam cells (Fredman and Spite 2013). A chronic inflammatory process is contributed to atherosclerosis. Moreover, activation of innate immunity system and chronic low-grade inflammation play important roles in all phases of atherosclerosis, including initiation, progression, and thrombotic complications (Libby 2012; Libby et al. 2010).

Toll-like receptors (TLRs) are a group of pathogen-associated molecular pattern receptors involved in innate immunity and pathogen recognition. To date, at least 13 TLRs have been discovered in mammals (O’Neill 2008). Toll-like receptor 4 (TLR4) belongs to the TLRs family and is expressed in cardiomyocytes, monocytoes, endothelial cells (Zarember and Godowski 2002) and cells of the central nervous system (Kielian 2006). TLR4 can ligate with not only the exogenous ligand lipopolysaccharide, in particular from gram-negative bacteria, but also several endogenous ligands, such as endogenous molecules released by injured tissues and necrotic cells. These molecules, called damage-associated molecular pattern molecules, induce the activation of a strong proinflammatory response through interaction with TLR4 (Molteni et al. 2016). Recognition of these ligands by TLR4 contributes to activation of signaling events that can elicit the pro-inflammatory cytokine release, lipid uptake, foam cell formation and even activate adaptive immune system (Cole et al. 2010). Stimulated TLR4 signaling results in the up-regulation of TNF-α production in macrophages, what is particularly important in terms of tissue inflammation (Nagai et al. 2013). Previous studies have showed that the expression of TLR4 was markedly enhanced in human atherosclerotic plaques and this augmentation occurred preferentially on macrophages and endothelial cells (Edfeldt et al. 2002; Vink et al. 2002; Otsui et al. 2007). An animal study also validated that *TLR4*-deficient mice sustained significantly smaller infarctions and lower level of inflammatory responses compared with wild-type control mice (Takeishi and Kubota 2009). These revealed a fact that *TLR4* might be an important susceptibility gene to CAD via its role in activation of innate immunity and inflammatory responses.

*TLR4* gene is located on chromosome 9 (9q32-q33) and consists of four exons and three introns. So far, 29 single nucleotide polymorphisms (SNPs) have been identified in the *TLR4* gene. With the use of SNPs as molecular markers, many genetic association studies were performed to investigate the associations of *TLR4* gene polymorphisms with the risk of CAD (Kolek et al. 2004; Balistreri et al. 2004; Incalcaterra et al. 2010; Zee et al. 2005; Koch et al. 2006; Nebel et al. 2007; Džumhur et al. 2012). However, results of these studies are not always consistent. Some of them showed that *TLR4* gene polymorphisms were associated with a lower risk of CAD (Kolek et al. 2004; Balistreri et al. 2004; Incalcaterra et al. 2010), while the others found no relationship between them (Zee et al. 2005; Koch et al. 2006; Nebel et al. 2007; Džumhur et al. 2012). All of above studies were performed in Caucasian populations and paid attention to two missense polymorphisms in the *TLR4* (Asp299Gly and Thr399Ile). Because both Asp299Gly and Thr399Ile are very rare in Chinese population, there are few relevant studies about the association of *TLR4* gene polymorphisms with CAD (Hang et al. 2004).

Considering TLR4 plays an important role in the pathogenesis of CAD, we hypothesized those common polymorphisms in the *TLR4* gene might predispose human to CAD. Therefore, the aim of this study was to explore the association between tag SNPs, which capture all the essential information about the *TLR4* gene locus, and CAD in a Chinese Han population.

## Results

### Characteristics of study subjects

The demographic and clinical characteristics of the 577 CAD patients and 517 control subjects are presented in Table 1. Compared with the control group, the CAD group had more smokers and more individuals with hypertension and with diabetes. Significant higher in age, WHR and TC was pronounced in the CAD group. There was no significant difference in gender, BMI, TG and prevalence of drinking status between the control and case groups.

**Table 1:** Baseline characteristics of the control and case group

Characteristic	Control (n = 517)	Case (n = 577)	*P*
Acute coronary syndrome, n (%)	–	266 (46.1)	–
Gender, n (%)			
Female	252 (48.7)	270 (46.8)	0.519
Male	265 (51.3)	307 (53.2)	
Age (years)^a^	63.75 ± 11.56	61.70 ± 12.75	0.080
BMI (kg/m^2^)^b^	24.02 ± 3.28	24.11 ± 2.73	0.691
WHR^a^	0.88 ± 0.09	0.93 ± 0.07	<0.001
Hypertension (%)	109 (31.6)	245 (56.1)	<0.001
Diabetes (%)	43 (12.5)	126 (23.8)	<0.001
Smoking (%)	84 (23.6)	199 (37.1)	<0.001
Drinking (%)	80 (22.5)	112 (21.2)	0.625
TC (mmol/L)^b^	4.64 ± 1.22	5.04 ± 1.06	<0.001
TG (mmol/L)^a^	1.74 ± 1.06	1.74 ± 1.14	0.611

^a^Median ± QR

^b^Mean ± SD

### *TLR4* polymorphisms and CAD risk

Three SNPs (rs1927907, rs1927911 and rs11536889) in *TLR4* were genotyped. All genotype distributions in both control and case groups were in Hardy–Weinberg equilibrium (*P* > 0.05, Table 2).

**Table 2:** Distribution of allele frequencies of SNPs in control and case groups

SNPs	Group	HWE	Allele numbers (%)		*χ* ^*2*^	*P*	*OR (95* % CI)*
			Major allele	Minor allele			
rs11536889	Control	0.454	820 (79.5)	212 (20.5)	0.506	0.477	1.104 (0.806, 1.512)
	Case	0.619	890 (78.2)	248 (21.8)			
rs1927907	Control	0.926	752 (73.3)	274 (26.7)	0.583	0.445	1.095 (0.813, 1.474)
	Case	0.542	849 (74.7)	287 (25.3)			
rs1927911	Control	0.713	425 (41.4)	601 (58.6)	0.161	0.689	0.910 (0.699, 1.184)
	Case	0.272	465 (40.6)	681 (59.4)			

* Adjustment for WHR, TC, history of hypertension and diabetes and smoking status

The allele frequencies of the 3 SNPs are listed in Table 3. There were no significant differences between healthy controls and CAD patients (*P* > 0.05). After adjustment for traditional risk factors including age, WHR, TC, history of hypertension and diabetes, and smoking status, no statistically association of the three SNPs with CAD was found between the case and control group (*P* > 0.05).

**Table 3:** Association of SNPs with CAD in different genetic models

SNPs	Model	Genotype	Control (%)	Case (%)	*χ* ^*2*^	*P*	*OR (95* % CI)*
rs11536889	Co-dominant	G/G	323 (62.6)	346 (60.8)	0.570	0.752	1.00
		G/C	174 (33.7)	198 (34.8)			1.055 (0.714, 1.558)
		C/C	19 (3.7)	25 (4.4)			1.293 (0.503, 3.326)
	Dominant	G/G	323 (62.6)	346 (60.8)	0.366	0.545	1.00
		G/C+C/C	193 (37.4)	223 (39.2)			1.100 (0.755, 1.603)
	Recessive	G/G+G/C	497 (96.3)	544 (95.6)	0.352	0.553	1.00
		C/C	19 (3.7)	25 (4.4)			1.270 (0.498, 3.235)
rs1927907	Co-dominant	G/G	276 (53.8)	320 (56.3)	0.702	0.704	1.00
		A/G	200 (39.0)	209 (36.8)			1.126 (0.764, 1.659)
		A/A	37 (7.2)	39 (6.9)			1.139 (0.534, 2.429)
	Dominant	G/G	276 (53.8)	320 (56.3)	0.701	0.402	1.00
		A/G+A/A	237 (46.2)	248 (43.7)			1.134 (0.783, 1.643)
	Recessive	G/G+A/G	476 (92.8)	529 (93.1)	0.049	0.824	1.00
		A/A	37 (7.2)	39 (6.9)			1.085 (0.517, 2.275)
rs1927911	Co-dominant	T/T	86 (16.8)	88 (15.4)	0.409	0.815	1.00
		C/T	253 (49.5)	289 (50.4)			0.844 (0.494, 1.442)
		C/C	174 (33.7)	196 (34.2)			0.797 (0.455, 1.398)
	Dominant	T/T	86 (16.8)	88 (15.4)	0.398	0.528	1.00
		C/T+C/C	427 (83.2)	485 (84.6)			0.824 (0.496, 1.370)
	Recessive	T/T+C/T	340 (66.3)	377 (65.8)	0.028	0.867	1.00
		C/C	173 (33.7)	196 (34.2)			0.926 (0.630, 1.361)

* Adjustment for WHR, TC, history of hypertension and diabetes and smoking status

We further compared the genotypic frequencies of each SNP between the two groups by using co-dominant, dominant and recessive genetic models. After adjustment for above confounding factors, the result showed that *TLR4* gene polymorphisms were not associated with the risk of CAD (*P* > 0.05, Table 3).

### Haplotype analysis

Three tag SNPs selected in our study were located in one haplotype block, and the magnitude of LD between each SNP was extremely high, with pair-wise *D*′ > 0.94. The haplotype analysis was performed to derive haplotypes specifically correlated with CAD susceptibility. As show in Table 4, although four common *TLR4* haplotypes (frequency >10 %) were found, there was no significant differences in the haplotype distributions between the control and case groups (*P* > 0.05).

**Table 4:** Haplotype frequencies of SNPs in control and case groups

Haplotype^a^	Control (%)	Case (%)	*χ* ^*2*^	*P*
rs1927911-rs1927907-rs11536889				
CGC	206 (20.2)	236 (21.2)	0.420	0.517
CGG	392 (38.5)	427 (38.3)	0.014	0.907
TAG	271 (26.6)	279 (25.0)	0.575	0.449
TGG	147 (14.4)	166 (14.9)	0.088	0.767

Global: *χ2* = 3.557, *df* = 6, *P* = 0.736

^a^Haplotypes with frequency >10 % were listed

### Quantitative trait analysis

The levels of TC and TG in the patient group were considered as quantitative trait to analyze their relationship with the 3 SNPs, respectively. However, neither of the two plasma lipid parameters showed association for the three SNPs (Table 5).

**Table 5:** Quantitative trait analysis for allelic association of the 3 SNPs with plasma lipid levels in patients with CAD

Lipid	SNPs	Major allele (%)	Minor allele (%)	Variance	*χ* ^*2*^	*P*
TC	rs11536889	890 (78.2)	248 (21.8)	2.002	2.448	0.118
	rs1927907	849 (74.7)	287 (25.3)	2.076	1.306	0.253
	rs1927911	464 (40.5)	682 (59.5)	2.050	0.005	0.944
TG	rs11536889	890 (78.2)	248 (21.8)	1.321	3.072	0.788
	rs1927907	849 (74.7)	287 (25.3)	1.331	0.309	0.578
	rs1927911	464 (40.5)	682 (59.5)	1.311	0.688	0.407

### Genotype association for hypertension and diabetes

Tables 6 and 7 analyzed the genotype association of the 3 SNPs with hypertension and diabetes in CAD patients. Neither of them showed association for the three SNPs.

**Table 6:** Genotype association of the 3 SNPs with hypertension in patients with CAD

SNPs	Major–major		Major–minor		Minor–minor		*χ* ^*2*^	*P*
	No (%)	Yes (%)	No (%)	Yes (%)	No (%)	Yes (%)		
rs11536889	45.9	54.1	33.8	68.2	44.0	56.0	2.072	0.355
rs1927907	40.7	59.3	48.1	51.9	42.9	57.1	2.202	0.333
rs1927911	42.0	48.0	43.9	46.1	46.8	43.2	0.417	0.812

**Table 7:** Genotype association of the 3 SNPs with diabetes mellitus in patients with CAD

SNPs	Major–major		Major–minor		Minor–minor		*χ* ^*2*^	*P*
	No (%)	Yes (%)	No (%)	Yes (%)	No (%)	Yes (%)		
rs11536889	78.4	21.6	71.0	29.0	75.0	25.0	3.448	0.178
rs1927907	74.8	25.2	76.7	23.3	85.7	14.3	2.084	0.353
rs1927911	76.8	23.2	74.0	26.0	81.2	18.8	1.871	0.392

## Discussion

In the present study, three tag SNPs (rs1927907, rs1927911 and rs11536889) in the *TLR4* gene were genotyped to investigate the association between *TLR4* gene polymorphisms and the risk of CAD. The minor allele frequencies of these SNPs in the control group were 26.7, 58.6 and 20.5 %, respectively, which were similar to that in the HapMap-CHB reference population. After systemic analysis, we found no evidence to support a significant association between *TLR4* gene polymorphisms and CAD susceptibility.

TLR4 is a type 1 transmembrane protein that mediates immune responses to both endogenous and exogenous ligands (O’Neill 2008). On binding of the specific ligands, TLR4 triggers signal transduction that induces the production and secretion of pro-inflammatory cytokines and chemokines through myeloid differentiation primary-response protein 88 (MyD88) or TIR domain-containing adaptor inducing IFN-β (TRIF) dependent signaling pathways, as appropriate (Seneviratne et al. 2012). Therefore, TLR4 plays a crucial role in the pathogenesis of CAD, while genetic variations within the gene have an important influence on the pathogenesis. A polymorphism Asp299Gly, resulting in amino acid exchange in the extracellular domain of the receptor, is associated with a blunted inflammatory response (Arbour et al. 2000). This might lead to altered risk of CAD. Previous studies have been performed to investigate the association of the polymorphism Asp299Gly with CAD susceptibility in Caucasian populations, albeit with conflicting results. Some of these studies showed that the minor G allele was associated with a reduced risk of CAD and acute coronary events (Kolek et al. 2004; Balistreri et al. 2004; Incalcaterra et al. 2010; Ameziane et al. 2003; Edfeldt et al. 2004; Kutikhin et al. 2016; Boekholdt et al. 2003; Holloway and Yang 2005), while the others found no relationship between them (Zee et al. 2005; Koch et al. 2006; Nebel et al. 2007; Džumhur et al. 2012; Golovkin et al. 2014; Morange et al. 2004; Hernesniemi et al. 2006; Lima-Neto et al. 2013). A meta-analysis indicated that Asp299Gly polymorphism was not associated with MI risk (Yin et al. 2014). However, the prevalence of Asp299Gly polymorphism is very low in Chinese population. Liu et al. (2012) and Lin et al. (2005) even failed to detect the presence of any variant for the SNP. So we did not investigate the role of the less common non-synonymous SNP in our study. Beside Asp299Gly polymorphism within *TLR4* gene, a study investigated the association between four new substitutions found by re-sequencing in the 5 V-proximal promoter region of the *TLR4* gene and acute myocardial infarction, but they did not find the association (De Staercke et al. 2007). We selected three tag SNPs for genetic analysis, including rs1927907, rs1927911 and rs11536889. Although any of them are not in exon, they are useful to find the disease-related variants because they capture abundant genetic information in the *TLR4* gene based on the CHB dbSNP database.

Two SNPs, rs1927907 and rs1927911, are located in intron. Introns are identified to have evolved to function as endogenous network control molecules, enabling direct gene–gene communication and multitasking of eukaryotic genomes, and are important in genetic expression and regulation (Mattick and Gagen 2001). A report regarding the relationship between the *TLR4* gene polymorphisms and late-onset Alzheimer’s disease (LOAD) showed that participants with AA genotype of rs1927907 had a significantly increased risk of LOAD (Chen et al. 2012). The minor allele C of rs1927911 was previously demonstrated to be associated with normal tension glaucoma (Takano et al. 2012; Shibuya et al. 2008), cancer (Zhang et al. 2013; Song et al. 2009) and pulmonary tuberculosis (Zaki et al. 2012). In our study, however, no statistically association was found between genetic variations in the rs1927907 and rs1927911 locus and the risk of CAD. The result is inconsistent with a relevant study performed in a Washington population, which revealed that minor allele C of rs1927911 was associated with a 12 % lower MI risk (Enquobahrie et al. 2008). The reason which can account for this contradictory result is the ethnic difference. The previous study was performed in a Caucasian population, while our study in a Chinese Han population.

The SNP rs1156889 is located in the 3′-UTR of the *TLR4* gene, where the sequence may influence mRNA stability, translation and localization and thereby regulate expression of the gene and interfere with the host immune system. In previous studies, the rs11536889 C was showed to be significantly associated with a high risk of emphysema (Ito et al. 2012) and prostate cancer (Kim et al. 2012). However, our study did not find any significant association between this SNP and CAD, relating to allele, genotype and haplotype analysis.

As a complex disease, there are overly confounding variables and genetic mechanisms that can lead to CAD. Analyzing the genetic variants can probably represent the first step to understand the pathophysiology of CAD, but can not explain the whole effect of TLR4 on CAD. Conditions of patients with CAD, such as hypertension, diabetes and plasma lipid levels, may influence the effect. Schneider S et al. had demonstrated an association between the TLR4 SNP rs4986790 genotype and age-dependant blood pressure increase in patients with coronary artery disease (Schneider et al. 2015). In order to take into consideration these factors affecting *TLR4* genetic analysis in CAD, We investigate the combined effect of *TLR4* and some common risk factors on the development of the disease. The results showed that there was no genotype association for plasma lipid levels, hypertension and diabetes. This suggested the plasma lipid levels, hypertension and diabetes did not influence the effect of TLR4 on CAD.

## Conclusion

The present study suggests that *TLR4* polymorphisms are not associated with CAD in the Chinese Han population. This study, to our knowledge, is the first attempt to assess the association of the three tag SNPs in *TLR4* gene with CAD in Chinese Han population and must contribute valuable information to the future relevant studies.

## Methods

### Study population

A total of 577 unrelated patients with CAD were recruited from the First Hospital of Jilin University between 2008 and 2012, including 266 patients with acute coronary syndrome. CAD was defined as the result of standardized coronary angiography and the diagnostic criteria of it was a ≥50 % stenosis in one or more main coronary arteries. All the angiographies were interpreted with the consensus from at least two independent cardiologists who were blinded to the results of the genetic analysis. Individuals were excluded if they suffered from congenital heart disease, cardiomyopathy, hepatic or renal disease and cancer. Control subjects (n = 517), residing in the same geographical area as the cases, were randomly selected from the persons who were through the routine checkup as part of annual body examination. They were classified as healthy controls based on the results of physical examination coupled with the absence of any reasons to suspect CAD.

Clinical data, including age, gender, body mass index (BMI), waist-to-hip ratio (WHR), hypertension, diabetes mellitus, smoking and drinking status, total cholesterol (TC) and triglyceride (TG), were obtained from all participants. Blood samples were collected in tubes containing EDTA and stored at −80 °C until extraction.

Every subject gave a written informed consent and was well told of the study protocol. The study was approved by the ethics committee of school of public health, Jilin University, Changchun, China.

### SNPs selection and genotyping

The data of reported *TLR4* SNPs for Chinese Han population in Beijing was obtained from the International HapMap Project (www.hapmap.org). Based on the criteria of minor allele frequencies (MAFs) ≥0.10 and linkage disequilibrium of *r*^*2*^ > 0.8, three SNPs were selected for genetic analysis. There are rs1927907 and rs1927911, located in the intron of *TLR4* gene, while rs11536889 in the 3′ untranslated region.

Genomic DNA was extracted from peripheral blood lymphocytes using a DNA extraction kit (TianGen, Beijing, China). Genotypes of SNPs were detected using Sequenom Mass-ARRAY system with genotyping success rate greater than 96 % for each SNP. The amplification primers and extension primers are listed in Table 8. For quality control, 30 random samples were genotyped repetitively together with others, and the concordance rate was 100 %.

**Table 8:** Amplification and extension primers used for genotyping *TLR4* gene

SNPs	Forward primers	Reverse primers	Extension primers
rs11536889	ACGTTGGATGTTTCCTGTTGGGCAATGCTC	ACGTTGGATGACCCCATTAATTCCAGACAC	TTTTTTCTCAATGATAACATCCACTC
rs1927907	ACGTTGGATGTAAGGTAGACCACCTCTCCC	ACGTTGGATGGGTATCCAGTGGATTGAAGA	GGAATTACATAAGAGACATTGTTTGA
rs1927911	ACGTTGGATGCATCACTTTGCTCAAGGGTC	ACGTTGGATGCAGACCTTCCTTAGTCATGG	AGAGTTTGACAACTGCATTCTTTTC

Continuous variables were expressed as median ± QR or mean ± SD and were compared by student’s t test or the Mann–Whitney U test, as appropriate. Categorical variables were expressed as counts (percentage) and were compared with Pearson *χ*^*2*^ test. The Hardy–Weinberg equilibrium for the genotypic distributions of SNPs was tested using the *χ*^*2*^ goodness-of fit test. The unconditional logistic regression models were performed to calculate the odds ratios (OR) and 95 % confidence intervals (CI). Three inheritance models (co-dominant, dominant and recessive) were defined and were applied to genotypic analysis. Genotype association for diabetes mellitus and hypertension was performed with *χ*^*2*^ test. The values of linkage disequilibrium (LD) between all pairs of biallelic loci were calculated using Haploview software (Version 4.2). Haplotypic analysis and quantitative trait tests were performed with the UNPHASED program (Version 3.0.12). A two-tailed *P* value <0.05 was considered to be statistically significant. Statistical analysis was carried out using SPSS 13.0 for Windows software.

## Abbreviations

BMI: body mass index
CAD: coronary artery disease
CI: confidence intervals
LD: linkage disequilibrium
LOAD: late-onset Alzheimer’s Disease
MAFs: minor allele frequencies
OR: odds ratios
SNPs: single nucleotide polymorphisms
TC: total cholesterol
TG: triglyceride
TLR4: Toll-like receptor 4
TLRs: Toll-like receptors (TLRs)
WHR: waist-to-hip ratio
